# Acoustic hologram optimisation using automatic differentiation

**DOI:** 10.1038/s41598-021-91880-2

**Published:** 2021-06-16

**Authors:** Tatsuki Fushimi, Kenta Yamamoto, Yoichi Ochiai

**Affiliations:** 1 R&D Center for Digital Nature, University of Tsukuba, Tsukuba, 305-8550 Japan; 2 Faculty of Library, Information and Media Science, University of Tsukuba, Tsukuba, 305-8550 Japan; 3 Graduate School of Library, Information and Media Studies, University of Tsukuba, Tsukuba, 305-8550 Japan; 4 Pixie Dust Technologies, Inc, Tokyo, 101-0061 Japan

**Keywords:** Applied physics, Acoustics, Engineering

## Abstract

Acoustic holograms are the keystone of modern acoustics. They encode three-dimensional acoustic fields in two dimensions, and their quality determines the performance of acoustic systems. Optimisation methods that control only the phase of an acoustic wave are considered inferior to methods that control both the amplitude and phase of the wave. In this paper, we present Diff-PAT, an acoustic hologram optimisation platform with automatic differentiation. We show that in the most fundamental case of optimizing the output amplitude to match the target amplitude; our method with only phase modulation achieves better performance than conventional algorithm with both amplitude and phase modulation. The performance of Diff-PAT was evaluated by randomly generating 1000 sets of up to 32 control points for single-sided arrays and single-axis arrays. This optimisation platform for acoustic hologram can be used in a wide range of applications of PATs without introducing any changes to existing systems that control the PATs. In addition, we applied Diff-PAT to a phase plate and achieved an increase of > 8 dB in the peak noise-to-signal ratio of the acoustic hologram.

## Introduction

Acoustic hologram is becoming an imperative part of a wide range of acoustics applications such as in the fields of medicine^1–3^, biology^4–8^, and engineering^9–11^. The reconstruction accuracy of the acoustic field from the hologram plays a significant role in determining the performance of the system. Thus, developing an acoustic hologram optimiser that can generate holograms with accurate field reconstruction is of significant interest of the field. Recent advancement of airborne phased array transducers (PATs) have enabled new applications such as airborne ultrasound tactile display (AUTD)^12,13^ and acoustic levitation^14–17^. Acoustic levitation in particular is used for digital fabrication^18,19^, display applications^20–25^ and sample holding in medicine^26,27^, physics^28,29^ and chemistry^30^. In all of these applications, acoustic holograms must generate a pressure control/focal point at a specified position. The control point is modulated at low frequencies to create haptic sensations that are sensible by human hands in an AUTD^12,13^. Marzo et al. demonstrated that the control points can be converted into levitation points by adding a twin-trap hologram in acoustic levitation^14,15^.

Generating an acoustic hologram for a single control point in space using PATs is trivial; however, it has been a significant challenge to generate a hologram that can create more than one control point in space. Multiple control points are becoming a necessary part of PAT applications, and low-quality acoustic hologram can lead to poor performances during practical use in applications. To address this challenge, Long et al. proposed Eigensolver and Tikhonov based regularisation in 2014^13^. Marzo & Drinkwater proposed the iterative back propagation (IBP) method in 2018^14^, and Plasencia et al. proposed GS-PAT in 2020^31^. GS-PAT and IBP are both modified versions of the Gerchberg-Saxton algorithm^32^. Most recently, Sakiyama et al.^33^ demonstrated acoustic hologram optimisation with the Levenberg–Marquardt algorithm (LMA); they examined the accuracy of ultrasonic stimulation in real and simulated environment. It should be noted that, while there are a number of hologram optimisers available within the research community, the usage of acoustic holograms to generate multiple focal points is a recent trend in the PAT community, and this field is yet to be developed. The listed acoustic hologram optimisers above are the most cited or considered state of the art in the field. LMA and IBP optimise only the phase of the transducer array, and GS-PAT and Eigensolver can optimise both the amplitude and phase of the acoustic hologram. Placensia et al.^31^ demonstrated that hologram optimisation methods with amplitude control (i.e. GS-PAT and Eigensolver) achieve higher-quality holograms than phase-only methods such as IBP, and that the Eigensolver method achieves the best optimised result because it seeks the global solution.

In this paper, we propose Diff-PAT, a phase-only, gradient-descent algorithm with automatic differentiation as a new platform for optimizing acoustic holograms. We demonstrate that in the most fundamental case of matching the output pressure to the target value; Diff-PAT achieve higher accuracy than the conventional Eigensolver and GS-PAT with both amplitude and phase control. Automatic differentiation is different from other differentiation operations (such as numerical differentiation and symbolic differentiation). This method calculates a derivative of a function at high accuracy by applying chain rules to each elementary operation of a given function. It is commonly used in machine learning. Our work was inspired by Peng et al.^34^, who demonstrated this automatic differentiation based method for optical holograms. In comparison to acoustic holograms, optical holograms have been studied for a longer period, and a number of hologram optimisation methods have been developed. However, Peng et al. demonstrated that the method using automatic differentiation outperforms conventional methods such as the Gerchberg-Saxton method. The challenge in optimising an optical or acoustic holograms is based on the fact that the loss function converts a complex number to a real number (non-holomorphic objective^35^). Whilst this function is challenging to differentiate analytically (the derivative of the function is not just a constant^35^), latest automatic differentiation packages^36–38^ can differentiate these functions with high precision^39^ and ease^34^. Diff-PAT does have multiple modes of implementation, and the gradient of the loss function does not necessarily need to be solved using automatic differentiation. Analytical and numerical differentiation can be just as effective for identifying the necessary information; however, it is cumbersome to analytically calculate the loss function (Wirtinger calculus is an advanced concept in methematics^34,40^), and the implementation with numerical differentiation is not as efficient as automatic differentiation (details in section on computational time). Considering the development cycle of the ultrasonic application, the implementation and execution time should be kept minimal to allow the user to test out different array configurations and loss functions to fit their purpose. Thus, we argue that automatic differentiation is the best mode of implementation for Diff-PAT considering its potential usage in early to late stages of development. Achieving a high-quality acoustic hologram without amplitude control in PATs represents a significant contribution to the literature, as it allows PAT controllers to retain a simple design. Furthermore, we demonstrated the versatility of Diff-PAT by employing it for the optimisation of a phase plate^41–44^. With regard to phase plates, the iterative angular spectrum approach (IASA) developed by Melde et al.^41^ is the standard method for optimizing acoustic holograms for arbitrary acoustic fields. We achieved enhanced performance by simply applying Diff-PAT to the optimisation process, without altering the fundamental design proposed by Melde et al^41^.

**Figure 1:**
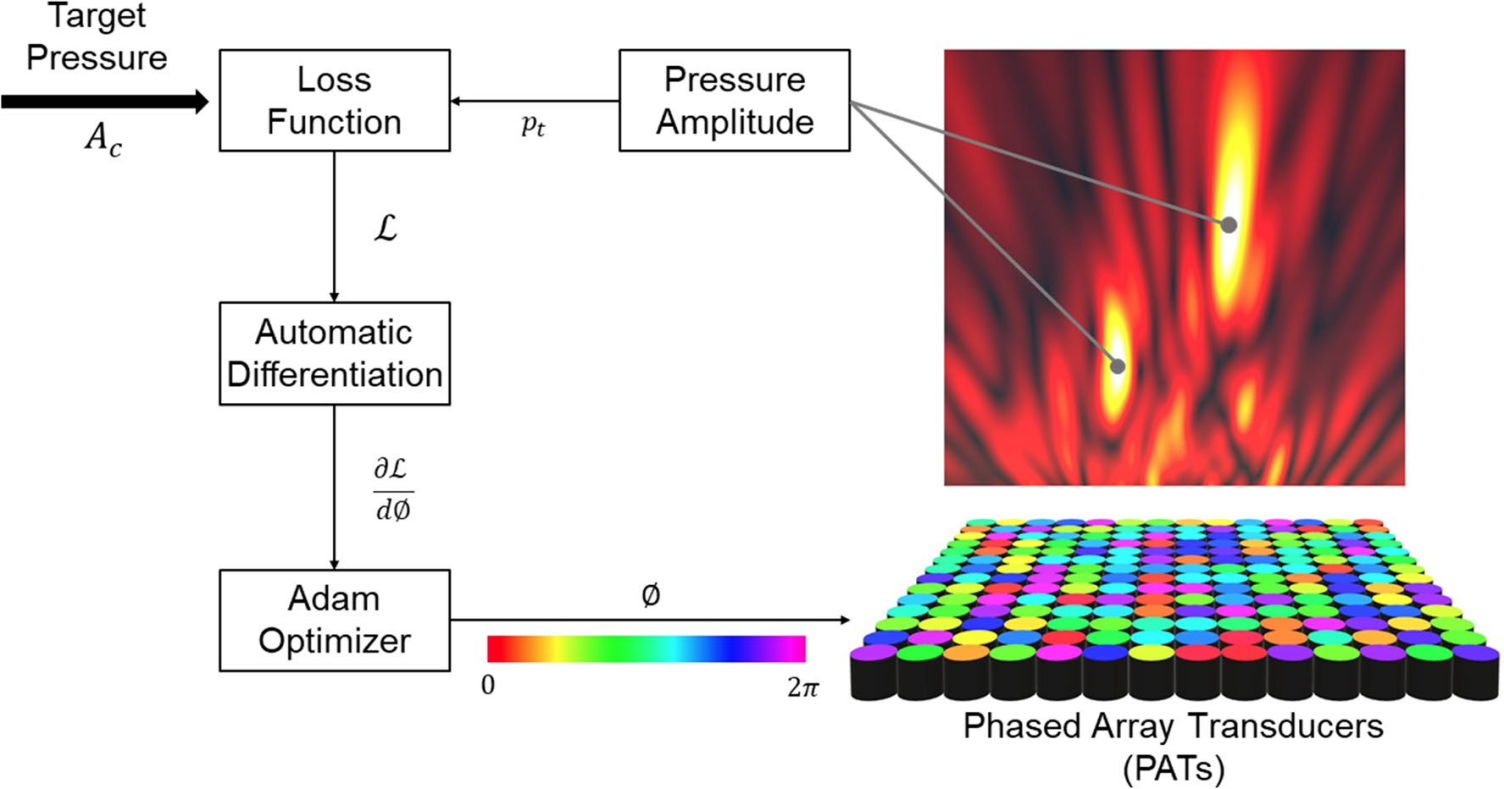
System overview for Diff-PAT. Loss function is evaluated by comparing the target and current acoustic amplitude, and automatic differentiation is used to calculate the derivative of the loss function. Image created using Microsoft Office PowerPoint 2019 (https://www.microsoft.com/en-ww/microsoft-365/powerpoint), Unity Version 2019.2.1f1 (https://unity.com/), and MATLAB 2020a Update 4 (https://www.mathworks.com/downloads/).

**Figure 2:**
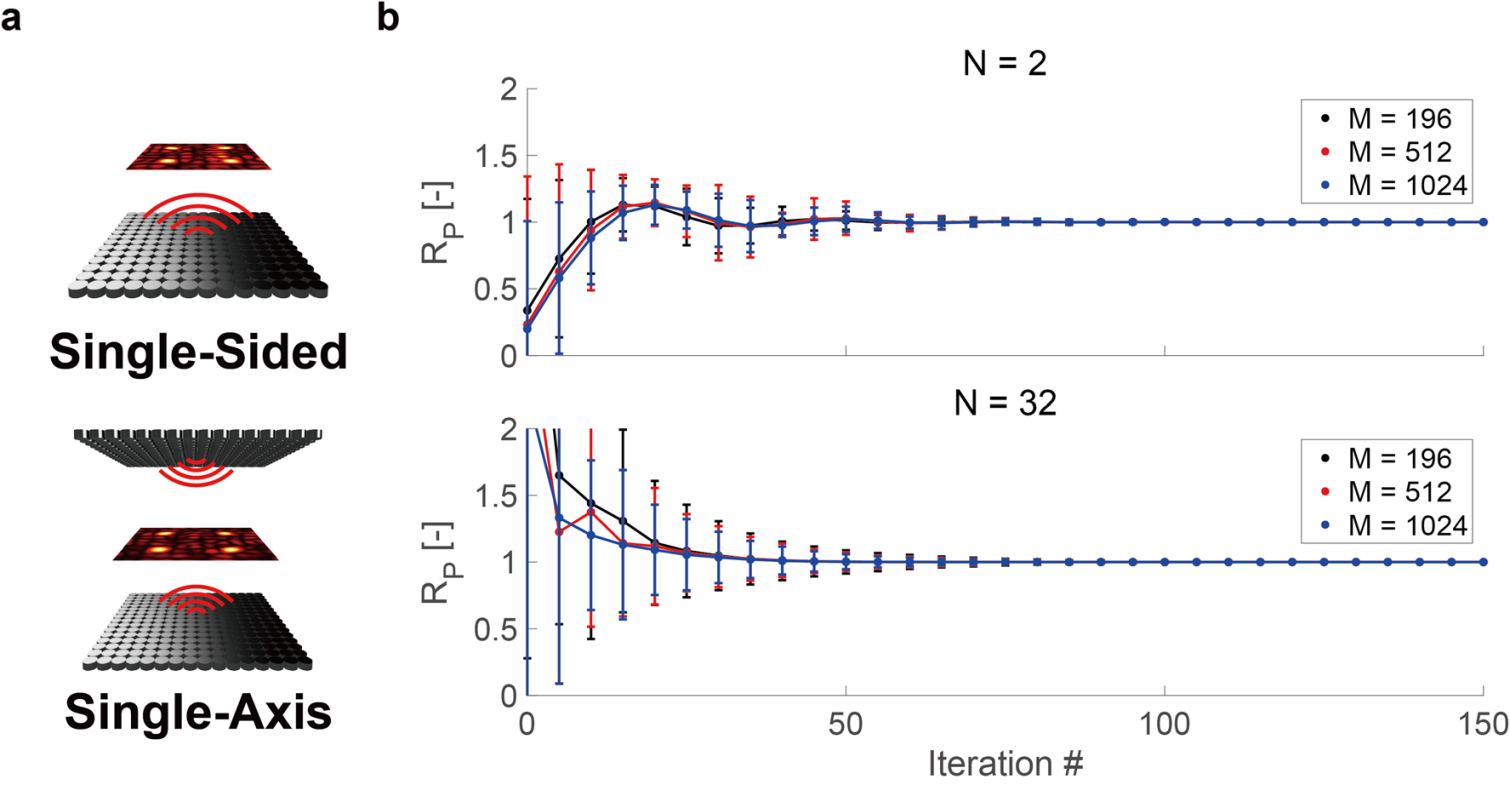
Array configuration and the convergence plot. (**a**) Configuration of single-sided and single-axis PATs. (**b**) Convergence of optimisation function $\mathcal{L}$ by the iteration number, control point number (N), and transducer number (M). The convergence is evaluated by ${{\varvec{R}}}_{{\varvec{p}}}$. The data point and the error bar show the mean and standard deviation of ${{\varvec{R}}}_{{\varvec{p}}}$, respectively, for the population of randomised 1000 sets of control points. Image created using MATLAB 2020a Update 4 (https://www.mathworks.com/downloads/), Adobe Illustrator 24.2.1 (https://www.adobe.com/products/illustrator.html), and Autodesk Fusion 360 ver. 2.0.9313 (https://www.autodesk.com/products/fusion-360/overview?term=1-YEAR).

The system overview of Diff-PAT is as shown in Fig. 1. We used JAX (ver. 0.2.5) on Python 3.6.9 to perform automatic differentiation and optimisation^38^. To identify a suitable acoustic hologram ($\phi_{n}$) for a given control point $x_{c}$, target amplitude $A_{c}$, and transducer position $x_{t}$, we adopted the Adam optimiser^45^, which enables efficient stochastic optimisation with only first-order gradients. Before the optimisation of hologram $\phi_{n}$, we defined the following optimisation problem and objective function :

$$ \begin{array}{*{20}c} {{\text{minimize}}} \\ {\phi_{n} } \\ \end{array} {\mathbf{\mathcal{L}}}\left( {\phi_{n} , x_{c} , A_{c} , x_{t} } \right) $$

where ${\mathbf{\mathcal{L}}}\left( {\phi_{n} , x_{c} , A_{c} , x_{t} } \right) = \mathop \sum \limits_{c = 0}^{C} (A_{c} - \left| {p_{t} \left( {\phi_{n} ,x_{c} ,x_{t} } \right)} \right|)^{2}$. $C$ is the total number of the control points, and $p_{t} \left( {\phi_{n} ,x_{c} ,x_{t} } \right)$ is the total acoustic pressure at $x_{c}$ (see “Methods” section for details on $p_{t}$). In order to evaluate the effectiveness of Diff-PAT in PATs applications, acoustic hologram was generated for three setups of arrays (see Fig. 2a for layout); (1) a single-sided square array of 14 $\times$ 14 transducers (M = 196), (2) two square arrays of 16 $\times$ 16 transducers (M = 512), which face each other with a separation distance of 0.2355 m (i.e. a single-axis configuration) and (3) a single-sided square array of 32 $\times$ 32 transducers (M = 1024). The single-sided array is a common arrangement for AUTDs, and the single-axis array is a popular configuration for acoustic levitation^14,46–48^. The single-sided square array with M = 1024 was included in the evaluation in order to allow for the increasing demand of 1000 + transducer PATs^18,49–51^. All results in this manuscript are based on numerical simulation; however, the utilised numerical models are well established in PATs, and the optimised results obtained from numerical simulation have been shown to translate well into physical experiments^13–15,21,31^. Some discrepancy between numerical and experimental results has been reported; however its effect is limited to specific application requirements such as accurate positioning^24,47,52^ and is otherwise is rarely considered. The initial phase estimate was randomly generated for all transducers. The gradient is calculated by reverse-mode automatic differentiation according to the computational graph of the objective function $\mathcal{L}$, and the phase is optimised by updating itself with the Adam optimiser on JAX. The Adam optimiser updates the current phase estimate according to the following^45^:

$$ \phi_{n} = \phi_{n - 1} - \alpha \cdot \frac{{\widehat{{m_{t} }}}}{{\left( {\sqrt {\widehat{{v_{t} }} + \varepsilon } } \right)}} $$

where $\widehat{{\mathrm{m}}_{\mathrm{t}}}=\frac{{\mathrm{m}}_{\mathrm{t}}}{1-{\upbeta }_{1}^{\mathrm{t}}}$ and, $\widehat{{\mathrm{v}}_{\mathrm{t}}}=\frac{{\mathrm{v}}_{\mathrm{t}}}{1-{\upbeta }_{2}^{\mathrm{t}}}$ are bias-corrected first and second raw moment estimates. ${\mathrm{m}}_{\mathrm{t}}={\upbeta }_{1}\cdot {\mathrm{m}}_{\mathrm{t}-1}+(1-{\upbeta }_{1})\cdot \frac{\partial \mathcal{L}}{\partial\upphi }$ , and ${\mathrm{v}}_{\mathrm{t}}={\upbeta }_{2}\cdot {\mathrm{v}}_{\mathrm{t}-1}+(1-{\upbeta }_{2})\cdot {\left(\frac{\partial \mathcal{L}}{\partial\upphi }\right)}^{2}$, where t is the iteration number, and ${\mathrm{m}}_{\mathrm{t}}$ and ${\mathrm{v}}_{\mathrm{t}}$ are both initially zero vectors. The required hyperparameters for the Adam optimiser, $\alpha$, ${\upbeta }_{1}$, ${\upbeta }_{2}$, and $\varepsilon$ were set as; 0.1, 0.9, 0.999, and $1\times {10}^{-8}$, respectively.

## Results and discussions

### Convergence rate of Diff-PAT

In the following section, the performance of Diff-PAT is evaluated. The best optimiser for PATs can be defined as the most versatile and accurate optimiser. In order to assess this, the performance of algorithms are evaluated using three array configurations and five levels of control point numbers. Control points are randomly generated using the process described in the “Methods” section, and this framework covers basic expectations of PATs in a wide range of applications and configurations available to date.

We show that the optimisation function converges quickly to the target value (evaluated by the ratio ${{\varvec{R}}}_{{\varvec{p}}}=\frac{\left|{{\varvec{p}}}_{{\varvec{t}}}({{\varvec{x}}}_{{\varvec{c}}})\right|}{{A}_{c}}$ between the target and current acoustic pressure amplitude), as shown in Fig. 2b (see Data Availability for results at each iteration). Increasing the number of control points (N) has a negative effect on the convergence rate of the solution, and the length of the error bar (which shows the standard deviation of ${{\varvec{R}}}_{{\varvec{p}}}$) increases. However, the increase in the number of transducers has a negligible effect on the convergence rate (Fig. 2b). We confirm that our algorithm converges sufficiently between 100 and 150 iterations in Fig. 2b, and the number of iterations was set to 150 in all evaluations of Diff-PAT in this study. The convergence rate of the algorithm can be further improved by hyperparameter tuning; however, this is beyond the scope of the current study. Here, the sum of squared error was chosen as the loss function; however, it could be evaluated using a number of different loss functions (such as Huber loss and sum of absolute error). The choice of loss function has an impact on the final results, and the sum of squared error gave the best results in the preliminary analysis. Further improvement in the loss function may be possible; however, such investigation is beyond the scope of the current study.

### Accuracy of Diff-PAT and comparison to conventional solvers

**Figure 3:**
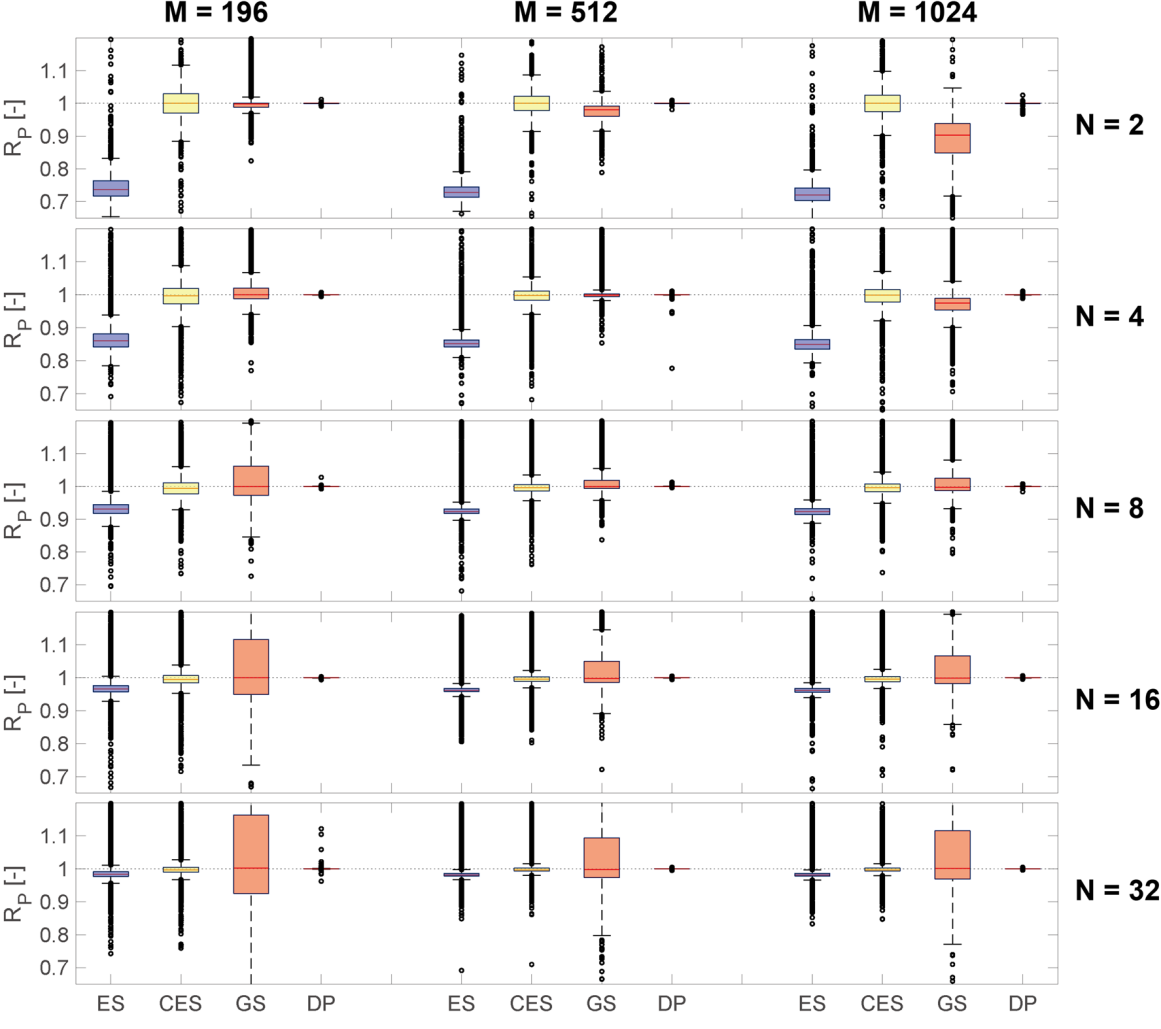
Box-and-whisker plot comparing ES (Eigensolver), CES (corrected Eigensolver), GS (GS-PAT), and DP (Diff-PAT) for different combinations of control points (N) and transducer numbers (M). The box shows the lower quartile, median, and upper quartile of the dataset (the total number of sample size or control points is 1000 $\times$ N) for each algorithm, and the maximum whisker length is set as 1.5 times the interquartile range. The black circles indicate outliers (i.e., values greater than the whisker length). Analysed using MATLAB R2020a Update 4 with Statistics and Machine Learning Toolbox Ver 11.7 (https://www.mathworks.com/downloads/). Image created using MATLAB R2020a Update 4 (https://www.mathworks.com/downloads/), subtightplot (ver. 1.2.0.0)^57^ and Adobe Illustrator 24.2.1 (https://www.adobe.com/products/illustrator.html).

The performance of Diff-PAT was compared with that of conventional algorithms in PATs which were made available by the authors of GS-PAT in C ++ ^31^. Here, we compare Diff-PAT with the Eigensolver, corrected Eigensolver, and GS-PAT. The results are shown in the box-and-whisker plot in Fig. 3. Each algorithm was tasked to optimise the acoustic hologram for the same dataset, which included 1000 sets of randomised control points and amplitude (GS-PAT's iteration number was set to 100, as in their study^31^). Raw data for Fig. 3 can be accessed as stated in the Data Availability section. For readers wishing to inspect the optimised acoustic pressure field in detail, the exemplary field from each optimiser is available in the supplementary material. The visual inspection of the acoustic pressure field shows that the target is achieved as specified in Diff-PAT. Furthermore, we performed a simple case study to demonstrate that the identified solutions by Diff-PAT are optimised solutions and that Diff-PAT strictly follows limits imposed by physical laws; details of which can be found in the supplementary material. The optimised phases from Diff-PAT were exported to a performance analyser developed by the author of GS-PAT, and the performance was analysed on it to independently verify our solution.

Interestingly, when the control number is small (N = 2), Eigensolver performs poorly (median values for M = 196, 512 and 1024 are 0.737, 0.728 and 0.721, respectively, in Fig. 3). This large deviation is considered to be caused by the tendency of the regularisation policy to “homogenise transducer's output rather than reconstruction accuracy”^31^. This performance can be improved by using the corrected Eigensolver method, and the median value improves to unity for M = 196, 512 and 1024 when N = 2.

In addition, the performance of Eigensolver and the corrected Eigensolver improves when the control point number increases from N = 2 to N = 32 in all types of arrays. Specifically, the interquartile (IQ) range value for the corrected Eigensolver method when N = 2 and M = 196 is 0.058; however increasing N to 32 improves the IQ range value to 0.015. We attribute this to the variability of the target amplitude in a given control point geometry. As the number of control points increases, the average difference between the minimum and maximum amplitude decreases. This is partially because of how the target amplitude is assigned when control points are randomly generated. The sum of the amplitudes assigned to the control points is always equal to a constant (see the “Methods” section), and the difference between the minimum and maximum amplitude for a given control point geometry decreases from 523, 283, and 70 Pa for N = 2, 8, and 32, respectively (M = 196 with corrected Eigensolver). This statement is supported by further analysis of the dataset of the corrected Eigensolver when N = 2 and M = 196. When the control point geometries lie between the IQ range, the average amplitude difference between the control points is 443 Pa. However, beyond the IQ range, the average amplitude difference between the control points increases to 603 Pa.

As shown in Fig. 3, the performance of GS-PAT decreases as the number of control points N increases. When N = 2 and M = 196, the IQ range is 0.012; however, when N is increased to 32, the IQ range increases to 0.238. By contrast, increasing the number of transducers to M = 512 improves the performance of GS-PAT, and the IQ range drops to 0.119 when N = 32 and M = 512. These results are consistent with the observations made by Placensia et al. who claimed that GS-PAT is comparable to Eigensolver up to N = 8 when M = 512. However, as the transducer number increases to M = 1024, the performance of GS-PAT drops again (IQ range increases to 0.089 and 0.146 for N = 2 and 32 when M = 1024).

Conventional solvers (i.e. Eigensolver, corrected Eigensolver, and GS-PAT) show a trade-off between the numbers of transducers and control points. In contrast, Diff-PAT outperforms all solvers in all cases evaluated in Fig. 3. Diff-PAT is robust against amplitude variability within the control point geometries and can consistently achieve the target amplitude despite the reduction in transducer numbers. In addition, while the Eigensolver type method and GS-PAT use both amplitude and phase control, Diff-PAT outperforms both methods with only phase control. It should be noted that Diff-PAT does have an increased background noise in comparison to the other algorithms (see acoustic pressure distributions from each solver in supplementary material). However, the increased background noise as obtained in this case study does not invalidate the effectiveness of Diff-PAT, as this is the direct results of the clearly set objectives in the loss function (i.e. only acoustic pressure at the target point is considered). Whether the increased background noise become an issue is largely dependent on each application. For example, in ultrasonic tactile displays increased background noise could lead to blunt haptic sensation, whereas in acoustic levitation, high background noise away from the control point may not arise as significant problem. It is difficult to ascertain the effect of the increased background noise, sole from the numerical simulation. Thus, the purpose of the paper is to set to introduce the effectiveness of Diff-PAT as the platform for optimizing acoustic systems, and we hope researchers from each field will develop suitable loss function which meet their purpose in their system.

### Comparing computational time of each solver

**Figure 4:**
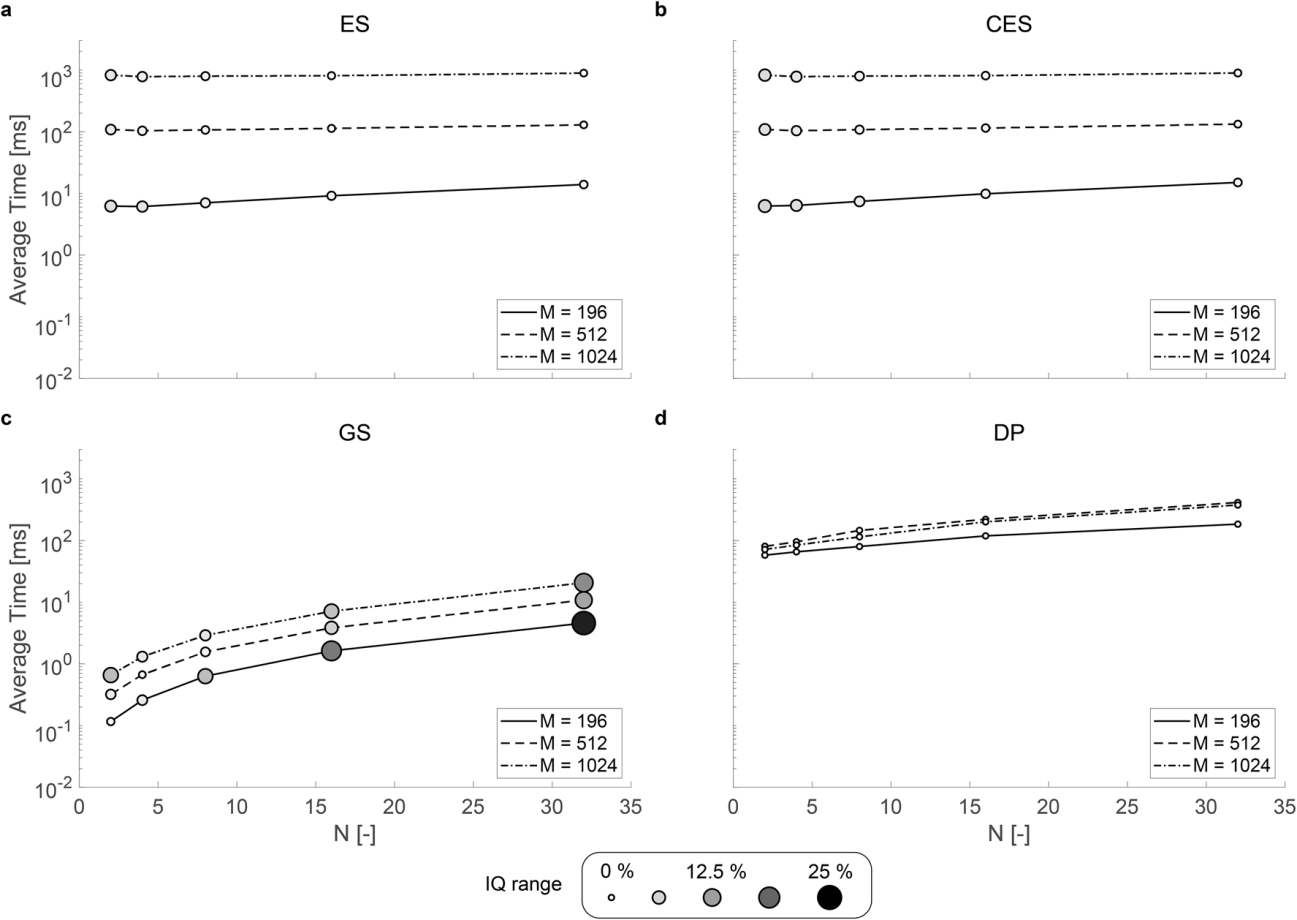
Comparing the average computational time for solving one geometry using each solver. The sample size is 1000 geometries, and solved the same dataset as in Fig. 3. The magnitude of IQ range from Fig. 3 is transposed to the marker size and darkness. Small, white marker indicates low IQ range; large dark marker indicates high IQ range. (**a**) Eigensolver (ES), (**b**) corrected Eigensolver (CES), (c) GS-PAT (GS) and (d) Diff-PAT (DP). (**a**)–(**c**) were based on a C +  + code, where (**d**) was run on Python. The performance was evaluated on the same desktop computer. Image created using MATLAB 2020a Update 4 (https://www.mathworks.com/downloads/) and Adobe Illustrator 24.2.1 (https://www.adobe.com/products/illustrator.html).

The computational time of the solver is insignificant for non-active PATs; however, some applications of PATs require consideration of the computational efficiency. Figure 4 compares the computational time of each solver, and all of the solvers were executed on the same high-end desktop computer (4.2 GHz Core i7-7700 K, 64 GB RAM). Eigensolver and corrected Eigensolver (Fig. 4a and b) both show similar trends in computational time, and an increase in control points (N) does not have a significant effect on their computational time. However, doubling the number of transducers results in a magnitude increase in the computational time for both Eigensolver and corrected Eigensolver (e.g. when N = 2, the computational time for Eigensolver is 6.2, 110, and 830 ms for M = 196, 512, and 1024, respectively). GS-PAT achieves the lowest computational time of all solvers in all of the conditions (lowest computational time is 0.12 ms with N = 2 and M = 196, and the highest computational time is 21 ms with N = 32 and M = 1024). The authors of GS-PAT have reported up to 17,000 geometries per second^31^, making it well suited for application requiring rapid calculations. However, as demonstrated in Figs. 3 and 4, GS-PAT is inaccurate in comparison to other solvers, and the regions in which GS-PAT can achieve both accuracy and speed is limited.

Diff-PAT does not have a computational efficiency that is as high as that of GS-PAT; however, its computational time is comparable to that of Eigensolver type optimiser with M = 512. When M = 512 and $\mathrm{N}\le 4$, Diff-PAT is faster than the Eigensolver type method and when M = 1024, Diff-PAT is faster than the Eigensolver method in all cases of N. In addition, Diff-PAT scales well with an increasing number of transducers as shown in Fig. 4d. Given that the demand for high transducer number PATs is increasing^18,49–51,53^: Diff-PAT is already more suited to optimise large transducer number PATs than an Eigensolver type method, both in terms of accuracy and computational efficiency. Furthermore, there are abundant applications of PATs where the accurate reconstruction of the acoustic field is prioritised over computational speed^18,20–22,54^. Diff-PAT can also be implemented using numerical differentiation as stated earlier. The accuracy of Diff-PAT with numerical differentiation is comparable to that of automatic differentiation; however, its computational efficiency is lower than that of automatic differentiation, as shown in the supplementary material. This is due to the fact that numerical differentiation is an O(n) process, whereas automatic differentiation scales better with the increasing number of variables in the loss function^39^.

### Application of Diff-PAT for Phase Plate

In this section, we further explore the versatility of Diff-PAT by employing Diff-PAT for the optimisation of a phase plate^41–44^ which is used underwater. It has a significantly higher number of elements than PATs^43^ and is well-suited for testing the capabilities of Diff-PAT. The IASA developed by Melde et al. is one of the most popular optimisation methods for phase plates. Similar to the GS-PAT and IBP methods, IASA also applies the Gerchberg–Saxton algorithm^32^. To create a phase plate version of Diff-PAT, the acoustic pressure field was assumed to be a plane wave^41^, and the angular spectrum approach^55^ was used to calculate the propagating acoustic field in the loss function (see “Methods” section for details).

**Figure 5:**
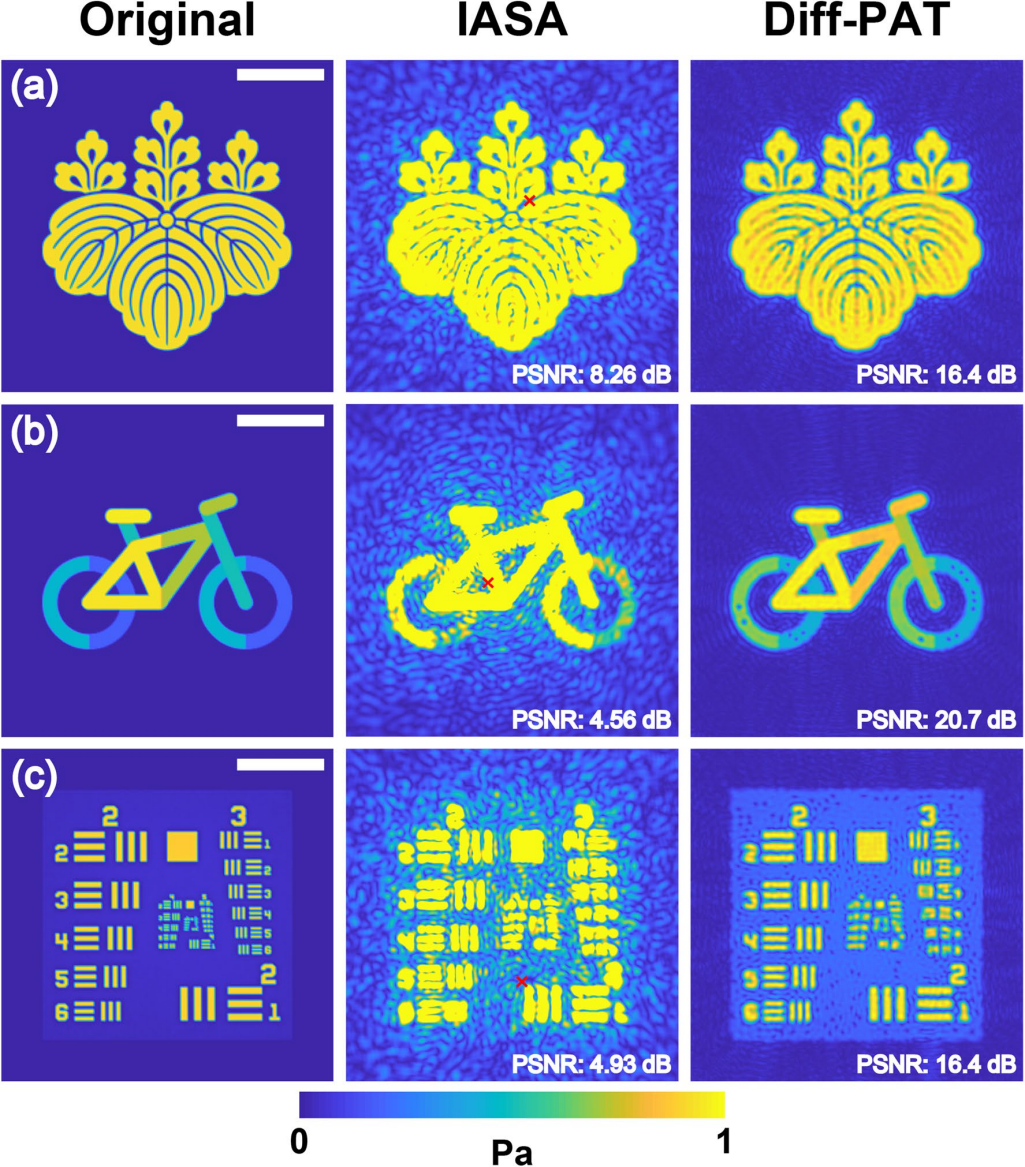
Comparison of acoustic holograms optimised using Diff-PAT and IASA. The resolution of the original image is 256 $\times$ 256 pixels. An underwater 2 MHz transducer with a 35 mm diameter was assumed, and the pixel size was set to 150 $\mathrm{\mu m}$. (**a**) Emblem of the University of Tsukuba (**b**) a bicycle (**c**) USAF 1951 resolution test chart. The scale bar shows 10 mm. The images above are of absolute acoustic pressure amplitude, and are not normalized. The colour axis is limited between 0 to 1 Pa since the original test image is defined as such. Due to this, the image of IASA is saturated. Red crosses in IASA show the point of maximum pressure amplitude deviation. For images (**a**)–(**c**) in IASA, the maximum pressure amplitude deviations are 1.65, 4.75, and 3.12 Pa, respectively. Image created using MATLAB R2020b Update 2 (https://www.mathworks.com/downloads/), k-Wave (ver. 1.3 http://www.k-wave.org/download.php)^56^and Adobe Illustrator 24.2.1 (https://www.adobe.com/products/illustrator.html).

Figure 5 compares the holograms optimised using IASA and Diff-PAT (see Supplementary Information for the acoustic holograms of IASA and Diff-PAT). The reconstruction accuracy of Diff-PAT is clearly higher than that of IASA. A simple visual inspection verifies that the acoustic hologram optimised using IASA, shown in Fig. 5a–c, has many artefacts and does not resolve the test image well (Fig. 5c). In contrast, the acoustic hologram optimised using Diff-PAT has a significantly improved image with minimal artefacts, as shown in Fig. 5a–b, and the USAF 1951 resolution test chart is clearly resolved in Fig. 5c. Quantitatively, the peak signal-to-noise ratio (PSNR) values for Diff-PAT are 16.4, 20.7 and 16.4 dB (Fig. 5a–c, respectively), and they are at least 8 dB higher the respective PSNRs of IASA. In practical applications of phase plates, these optimised acoustic holograms are encoded and fabricated into a plate^41^ using a 3D printer. As shown in the supplementary material, Diff-PAT’s acoustic hologram has a spatially higher frequency component in comparison with IASA. This could potentially cause issues during manufacture of the phase plates. The effect of such manufacturing tolerances was evaluated, as presented in the supplementary material, and it was found that the PSNR drops by 3 dB when the standard deviation of normally distributed manufacturing tolerance drops to σ≈20%. If manufacturing tolerances become an issue, some weighted regularization term or a low pass filter can be added in the loss function to reduce the high frequency component of the acoustic hologram. The detailed view of acoustic pressure distribution demonstrates that Diff-PAT achieves this high accuracy in the region of interest (ROI) by diffracting the acoustic wave outside the ROI as shown in the supplementary material. The improved accuracy of Diff-PAT with significantly reduced artefacts in the ROI can enhance performance of a wide range of acoustic systems used in medicine^1–3^, biology^4–8^, and engineering^9–11^ applications.

In conclusion, we presented Diff-PAT, which optimises only the phase of an acoustic hologram using automatic differentiation. We demonstrated the effectiveness of Diff-PAT as a new platform for optimising acoustic holograms in PATs and phase plates. In addition, contrary to common belief, the phase-only optimiser can achieve better accuracy than an optimiser with both amplitude and phase control in the simplest test case for acoustic holograms. For PATs with high transducer numbers (M = 1024), Diff-PAT is more suitable than Eigensolver type methods, both in terms of accuracy and computational efficiency. In future, this improved acoustic hologram optimiser will improve the performance of many applications by providing higher quality acoustic holograms. In addition, this study, along with that of Peng et al.^31^, demonstrated the effectiveness of implementing automatic differentiation in the context of hologram optimization. We hope that these results will further facilitate the use of Diff-PAT platform in a wide range of fields and applications in acoustics.

## Methods

### Total acoustic pressure calculation

The total pressure was evaluated by ${p}_{t}=\sum_{m=0}^{M}\frac{{P}_{ref}}{d\left({x}_{c}, { x}_{t}\right)}D\left(\uptheta \right){e}^{j(kd\left({x}_{c},{ x}_{t}\right)+{\upphi }_{n,m})}$ where $M$ is the total number of transducers, ${P}_{ref}$ is the reference pressure amplitude; $d\left({x}_{c}, {x}_{t}\right)$ is the Euclidean distance between ${x}_{c}$ and ${x}_{t}$, and $D\left(\theta \right)= \frac{2{J}_{1}(krsin\left(\uptheta \right))}{krsin\left(\uptheta \right)}$ is the far-field directivity function for the piston source based on the angle, $\uptheta$. Moreover, $\uptheta$ is evaluated as the angle between the transducer normal and ${x}_{c}$; $r = 5$ mm is the transducer radius, and ${J}_{1}$ is the Bessel function of the first kind of order 1. $k=\frac{2\uppi f}{{c}_{0}}$ is the wavenumber given the acoustic frequency, $f = 40 \mathrm{kHz}$ and the speed of sound is ${c}_{0}$. ${P}_{ref}$ and ${c}_{0}$ are assumed to be 1.98 Pa at 1 m (12 V Pk-Pk^25^) and $346 {\mathrm{ms}}^{-1}$, respectively. In the implementation of Diff-PAT, the acoustic pressure calculation is split into pre-calculated (A) and re-calculated (B) components: ${\mathrm{p}}_{\mathrm{t}}=\sum_{\mathrm{m}=0}^{\mathrm{M}}\mathrm{AB}$, where $\mathrm{A}=\frac{{\mathrm{P}}_{\mathrm{ref}}}{\mathrm{d}\left({\mathrm{x}}_{\mathrm{c}}, {\mathrm{ x}}_{\mathrm{t}}\right)}\mathrm{D}\left(\uptheta \right){\mathrm{e}}^{\mathrm{j}(\mathrm{kd}\left({\mathrm{x}}_{\mathrm{c}},{\mathrm{ x}}_{\mathrm{t}}\right))}$, and $\mathrm{B}={\mathrm{e}}^{\mathrm{j}({\upphi }_{\mathrm{n},\mathrm{m}})}$. Given the transducer properties do not change, part A does not change at all, and it allows significant increase in computational efficiency. In addition, neither TensorFlow 2.3.0 nor JAX 0.2.5 currently supports differentiation of Bessel functions of the first kind of order 1, and these terms cannot be included within the computational graph of automatic differentiation.

### Random generation of control points and amplitude

Control points, ${x}_{c}$ are randomly generated such that $x$, $y$, and $z$ coordinates are within the region of interest (ROI), i.e. $[-0.05, 0.05]$ m from the centre of the array. For single-sided arrays, the z-axis centre is set at $z=0.1$ m. The physically achievable pressure amplitude was predetermined by making a singular focal point ($\phi =-\frac{2\uppi f}{{c}_{0}}\left[d\left({x}_{r},{x}_{t}\right)-d\left(0,{x}_{r}\right)\right]$) at the vertex of ROI, ${x}_{r}$ (8 points in total). The average acoustic pressures around the vertices for single-sided (M = 196), single-axis (M = 512), and large single-sided (M = 1024) PATs are 1512, 3812, and 4121 Pa, respectively. Amplitude ${A}_{c}$ for each control point is randomly assigned such that the minimum pressure amplitude is 10 Pa, and the amplitude at each control point sums up to the average acoustic pressure for the single focus of each transducer array. This target amplitude assignment method is technically inaccurate as it assumes the acoustic amplitude to be conserved (i.e. acoustic power/energy should be conserved). However, the assignment of target via acoustic energy is impractical as it requires assignment of both target pressure and velocity field. Thus, this target assignment method is employed in this manuscript, and it is considered appropriate since the optimizer can follow the targets specified.

### Application of Diff-PAT to the phase plate

For the optimisation of the phase plate, an underwater transducer with a resonance frequency of 2 MHz, diameter of 35 mm, and input amplitude of 1 Pa was assumed at the surface. The input pressure field was assumed to be a plane wave, and the initial estimate of the phase was set to zero for all elements. The speed of sound in water was assumed to be 1480 ${\mathrm{ms}}^{-1}$, and the pixel size was assumed to be 150 $\mathrm{\mu m}$. For simplicity, the acoustic transmission loss through the plate was considered negligible, and the angular spectrum was solved using methods described by Zeng & McGough^55^ (following the implementation of k-Wave^56^). Both IASA and Diff-PAT were programmed in Python 3.6.9, and the phase plate version of Diff-PAT used Tensorflow^36^ (ver. 2.3.0) to differentiate the loss function automatically. We also used the Adam optimiser in TensorFlow with the same hyperparameter setting as in the PAT version. IASA was calculated by following the steps described by Melde et al.^41^, using the angular spectrum approach proposed by Zeng & McGough^55^. The propagation distance from the transducer to the image plane was 20 mm, and the iteration number for the optimiser (both IASA and Diff-PAT) was set to 200. The loss function was $\mathop \sum \limits_{x}^{N} \mathop \sum \limits_{y}^{N} \left| {A_{c} \left( {x,y} \right) - \left| {p\left( {x,y} \right)} \right|} \right|$, where $A_{c} \left( {x,y} \right)$ represents the target image with a screen resolution (N) of 256 $\times$ 256 pixels. The optimised acoustic hologram was exported from Python and the resultant acoustic pressure fields shown in Fig. 5 were calculated using k-Wave (ver. 1.3 *angularSpectrumCW*)^56^ on MATLAB. Finally, the PSNR was calculated using the MATLAB Image Processing Toolbox.

## Supplementary Information

Supplementary Information.

## Data Availability

The data that support the findings of this study are available within this article. The data for transducer arrangements, control point geometries and amplitude, output phase by Diff-PAT, and the results from all solvers are available in Zenodo at 10.5281/zenodo.4906351.

## Code availability

The program code for optimisation and analysis is also made available at the link provided in Data Availability.
