# Supplementary material for: Acoustic hologram optimisation using automatic differentiation
Source: Sci Rep. 2021 Jun 16;11:12678. doi: 10.1038/s41598-021-91880-2 (PMC8209099; doi:10.1038/s41598-021-91880-2)
Supplement: Supplementary file 1 — Supplementary Information. [file 41598_2021_91880_MOESM1_ESM.pdf]

Acoustic Hologram Optimisation Using Automatic Differentiation

Tatsuki Fushimi[1, 2, 5]*, Kenta Yamamoto[1,3, 5], Yoichi Ochiai[1,2,4]

[1] R&D Center for Digital Nature, University of Tsukuba, Tsukuba 305-8550, Japan

[2] Faculty of Library, Information and Media Science, University of Tsukuba, Tsukuba 305-8550, Japan

[3] Graduate School of Library, Information and Media Studies, University of Tsukuba, Tsukuba 305-8550, Japan

[4] Pixie Dust Technologies, Inc., Tokyo 101-0061, Japan

[5] These authors contributed equally: Tatsuki Fushimi, Kenta Yamamoto.

* Corresponding author email: tfushimi@slis.tsukuba.ac.jp

1. Visual Comparison between Acoustic Pressure Field Optimised by each Optimiser

Visualization of optimised acoustic hologram when N = 2, M = 512, sample index = 1, are as shown in supplementary Table 1. Top row is for the bottom arrays, and the bottom row is for top array.

Optimiser	Acoustic Pressure Field
ES	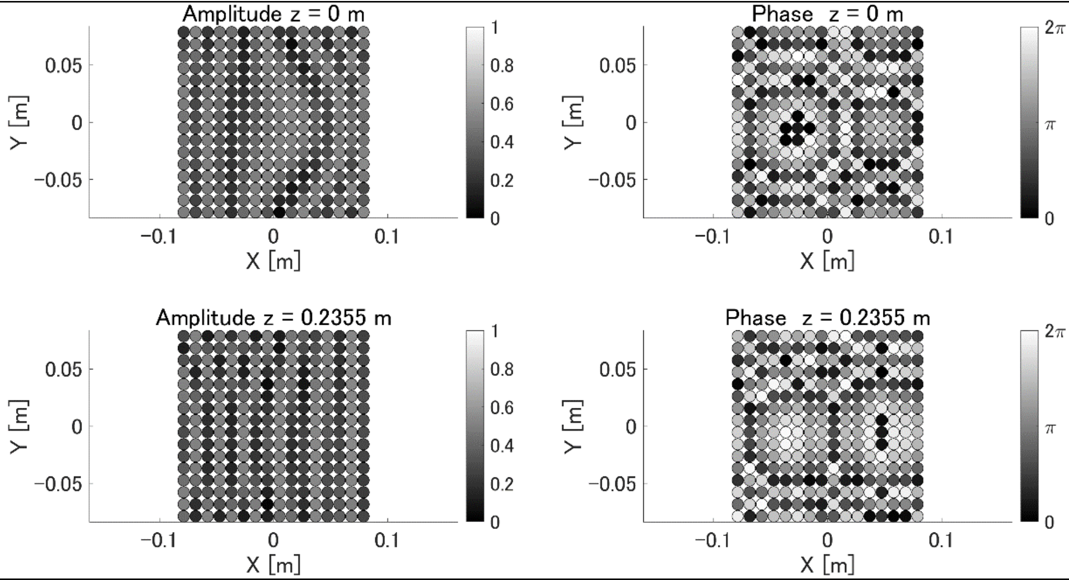
CES	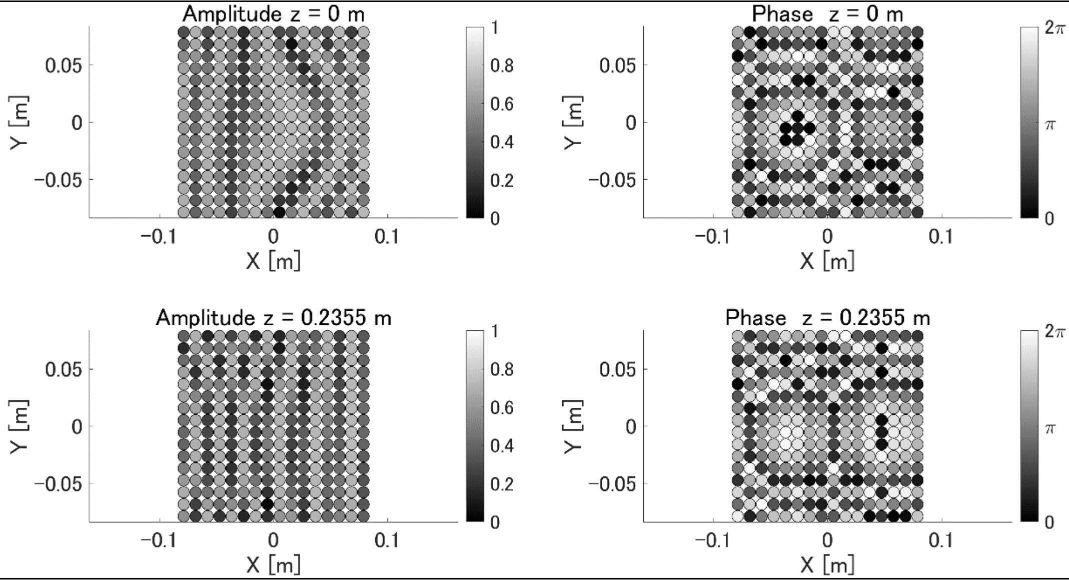

GS	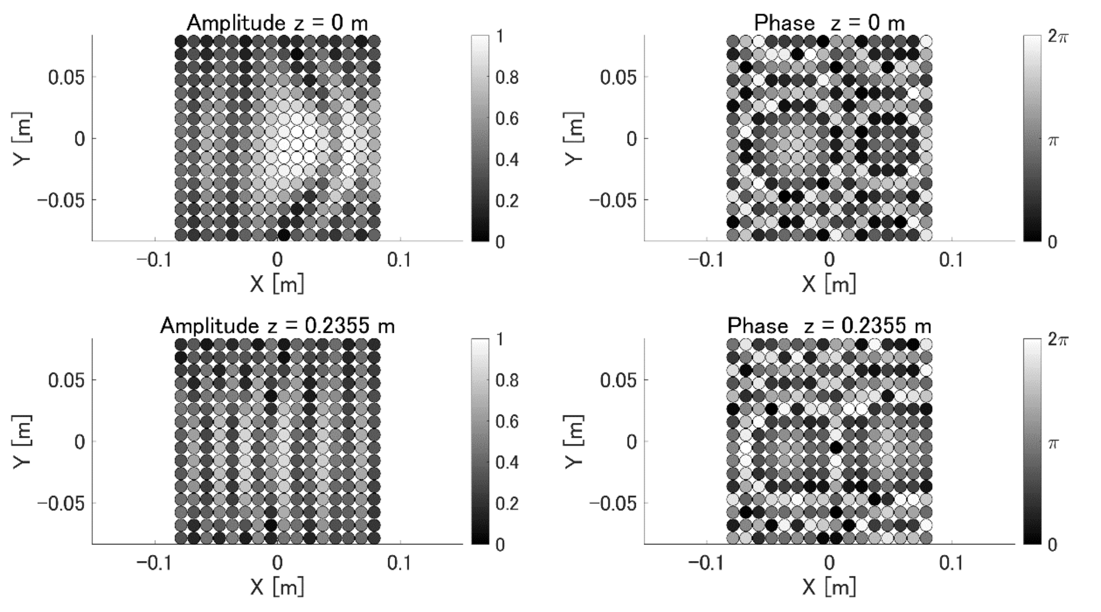
DP	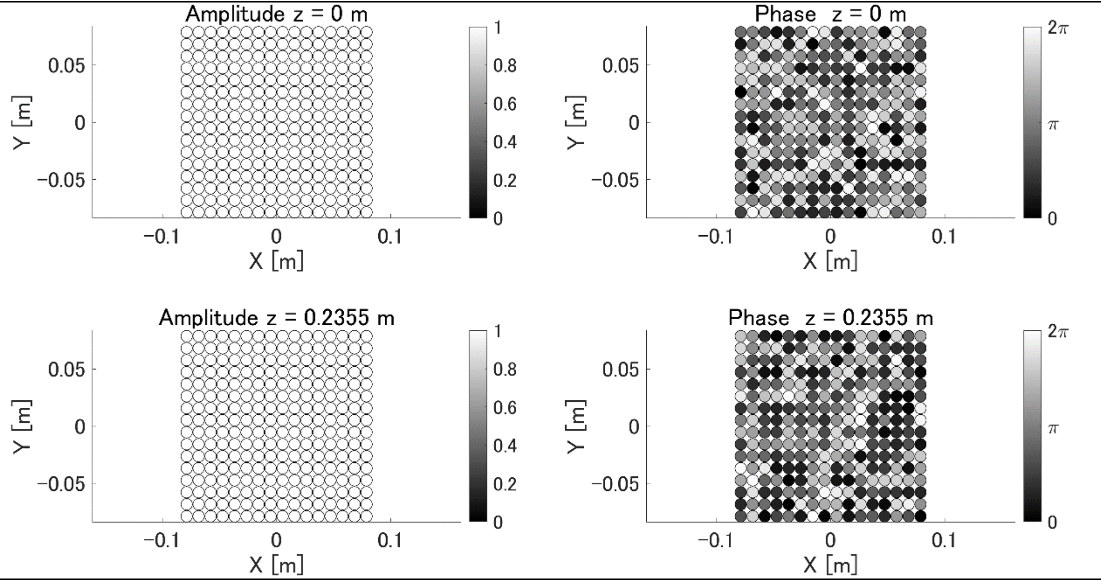

Supplementary Table 1: Acoustic hologram from each optimiser. Images created using MATLAB R2020b Update 2 (https://www.mathworks.com/downloads/).

The corresponding acoustic pressure field are shown below. Left column is for the first control point, and right column is for the second control point. The position of the control point is indicate with a black cross.

<table>
<tr><th>Optimiser</th><th>Acoustic Pressure Field</th></tr>
<tr><td>ES</td><td>
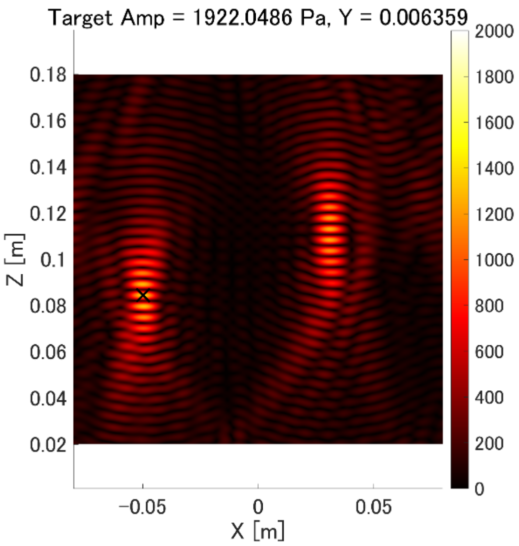

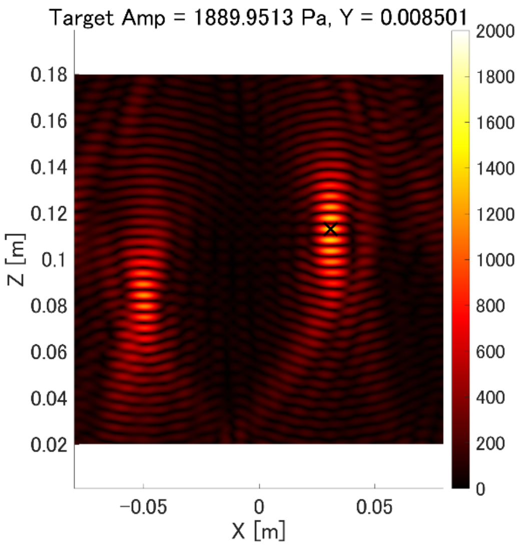

</td></tr>
<tr><td>CES</td><td>
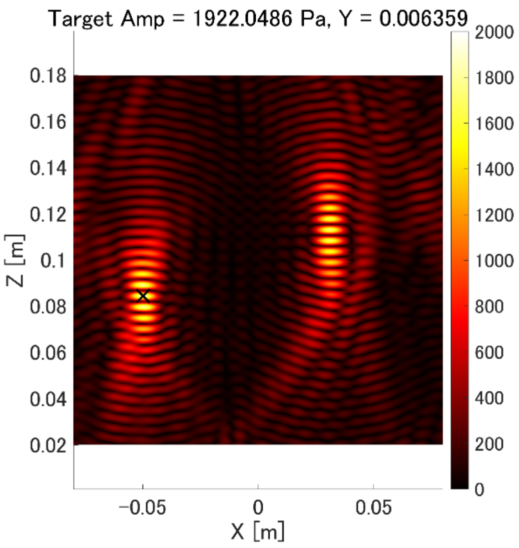

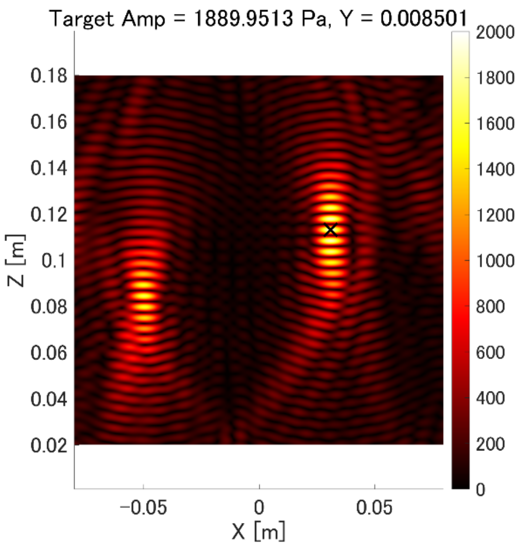

</td></tr>
</table>

<table>
<tr><td>GS</td><td>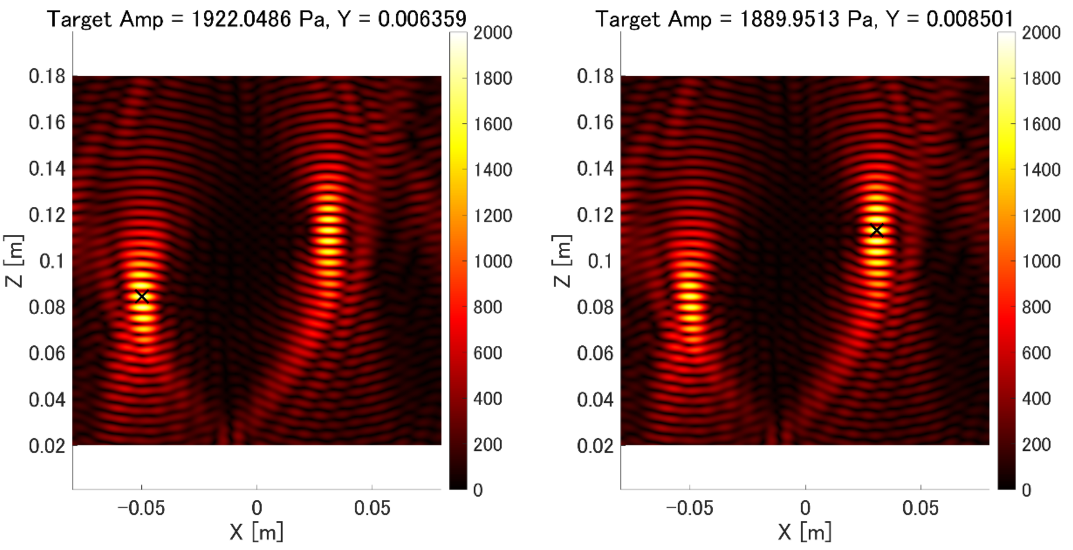
</td></tr>
<tr><td>DP</td><td>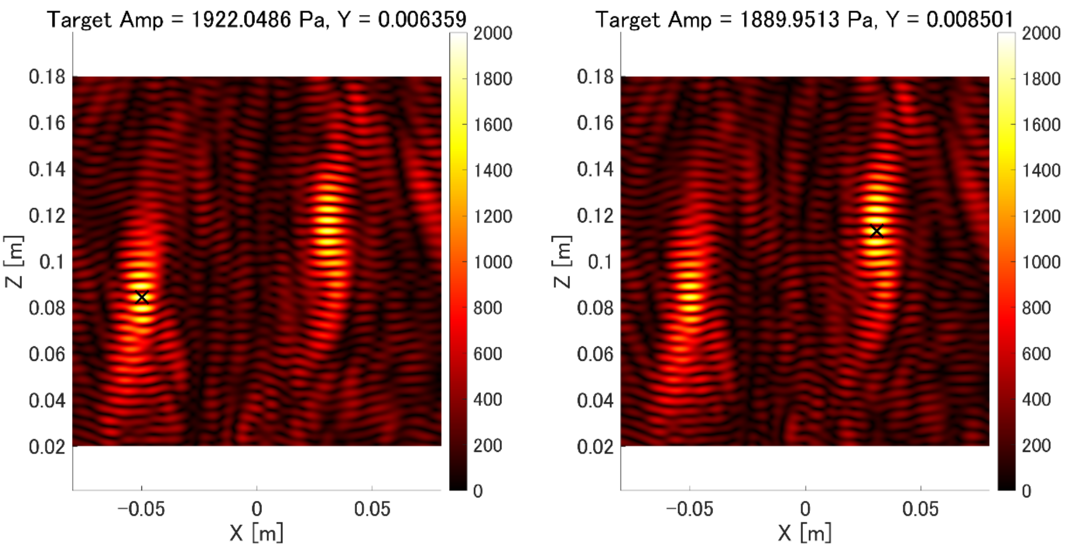
</td></tr>
</table>

Supplementary Table 2: Corresponding acoustic pressure field from each optimiser. Images created using MATLAB R2020b Update 2 (https://www.mathworks.com/downloads/).

Supplementary Table 2 visually confirms that the target amplitude is achieved at target point.

2. Comparison of analytical vs Diff-PAT solution

To establish whether Diff-PAT is finding impossible solutions or not, a comparison between analytical solution and optimised solution was made. Firstly, the theoretical maximum amplitude for a single focus was calculated using (for M = 512 transducer array):

$$\phi = -\frac{2\pi f}{c_0}[d(x_r, x_t) - d(0, x_r)]$$

Secondly, the Diff-PAT was tasked to achieve 80, 90, 110 and 120% of the theoretical pressure amplitude at each focal point. The result is as shown in Suppletory Figure 1.

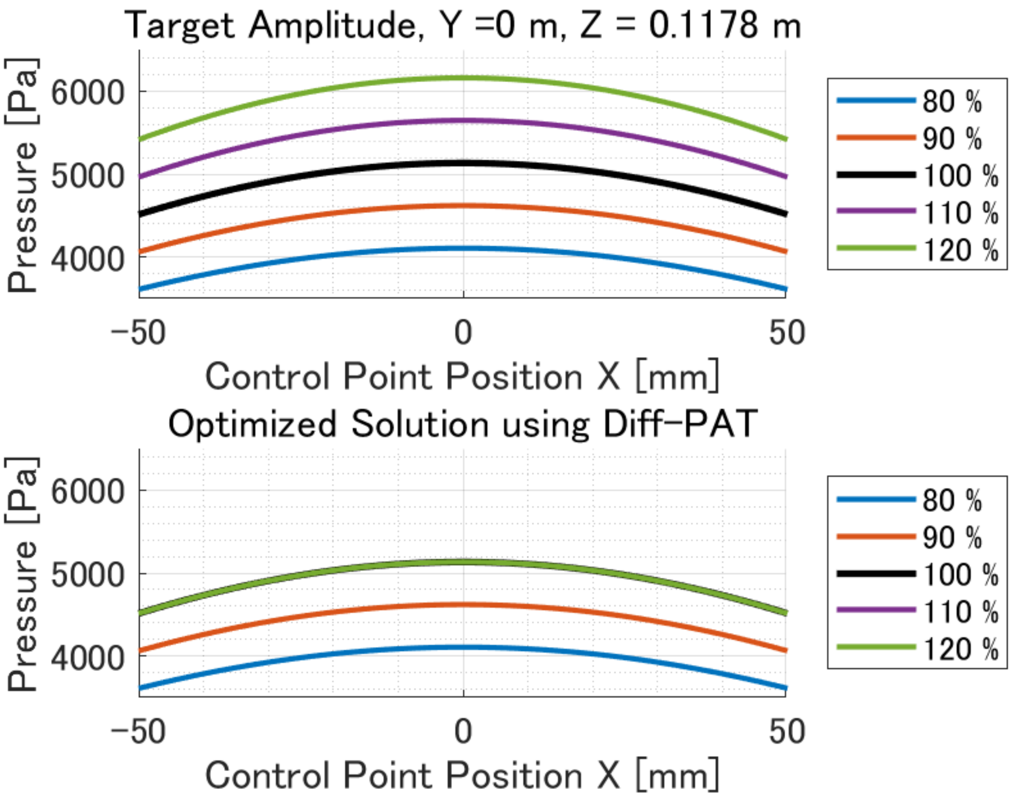

Supplementary Figure 1: Comparison of theoretical maximum acoustic amplitude and optimised solution of Diff-PAT. Images created using MATLAB R2020b Update 2 (https://www.mathworks.com/downloads/).

Supplementary Figure 1 clearly demonstrates that although Diff-PAT attempt to minimize the loss function, it is strictly bound by what is physically possible.

3. Comparison of Loss functions

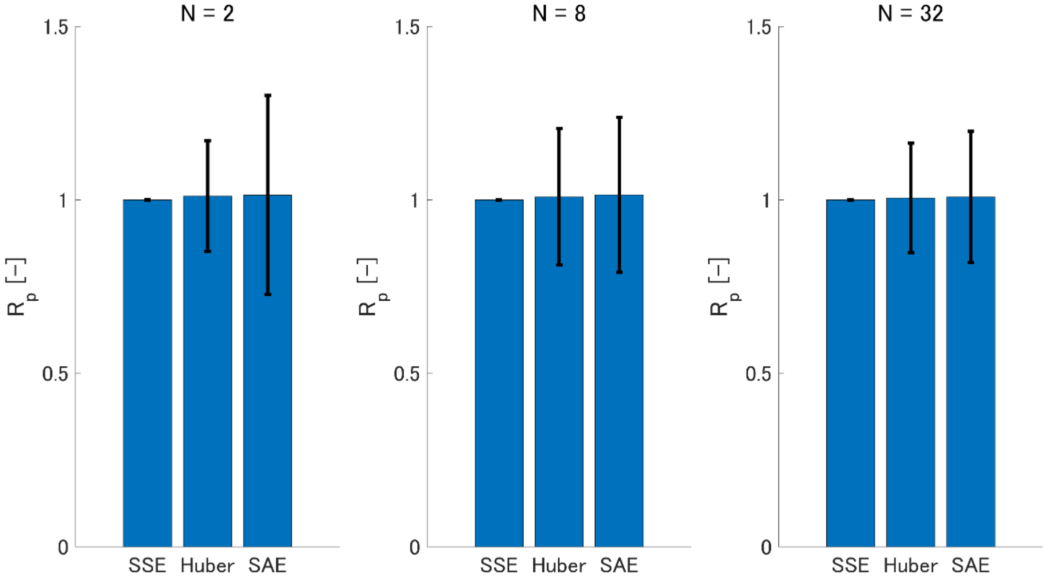

Supplementary Figure 2: Comparison of different loss function. Images created using MATLAB R2020b Update 2 (https://www.mathworks.com/downloads/).

Three types of loss functions were compared

$$\varepsilon = A_c - |p_t(\phi_n, x_c, x_t)|$$

- Sum of Squared Error: $\mathcal{L} = \sum_{c=0}^{C} \varepsilon^2$
- Sum of Absolute Error: $\mathcal{L} = \sum_{c=0}^{C} |\varepsilon|$
- Huber Loss function: $\mathcal{L} = \sum_{c=0}^{C} K$

$$K = \begin{cases} 0.5(\varepsilon^2) & |\varepsilon| < 1 \\ |\varepsilon| - \frac{1}{2} & otherwise \end{cases}$$

These loss functions were evaluated for N = 2, 8 and 32 when M = 512, and iteration number was set to 150. The results are as shown in Supplementary Figure 2. All of the loss function has mean R_p of 1, but the standard deviation for sum of absolute error and Huber loss function is greater than sum of squared error.

4. Numerical Differentiation version of Diff-PAT

Diff-PAT can be implemented using different modes of differentiation, and we implemented the numerical differentiation version for Diff-PAT using Julia. Forward differentiation scheme was used to evaluate the gradient of the loss function:

$$f'(x) = \frac{f(x+h) - f(x)}{h}$$

and the hyperparameter setting for Adam was kept the same, and the iteration number is the same 150. The computational time of Diff-PAT with numerical and automatic differentiation version was compared, and the results are as shown in Supplementary Figure 3.

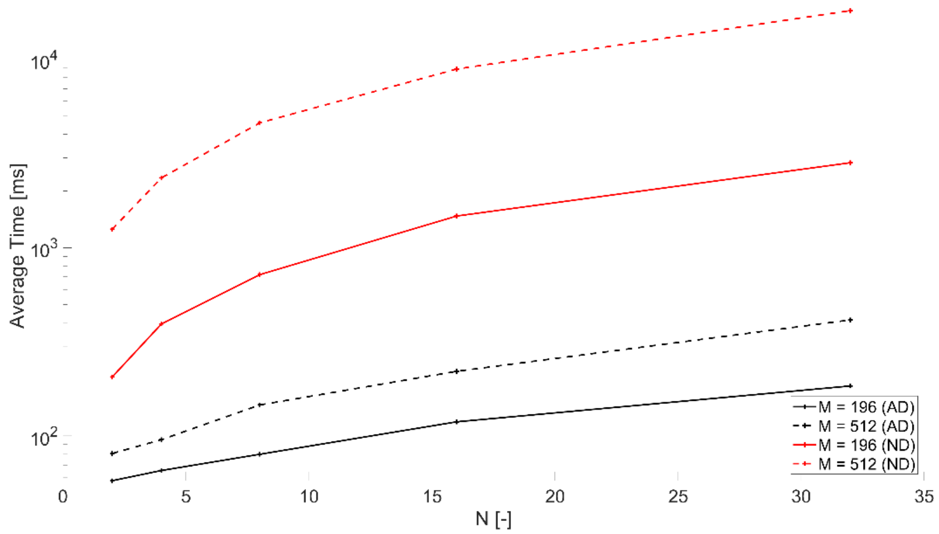

Supplementary Figure 3: Comparing the execution time of numerical differentiation (ND) and automatic differentiation (AD) version of Diff-PAT. Images created using MATLAB R2020b Update 2 (https://www.mathworks.com/downloads/).

Same high-end desktop computer (4.2 GHz Core i7-7700K, 64 GB RAM) was used for the evaluation, and the AD is shown to have higher efficiency than numerical differentiation version.

5. Corresponding Acoustic Holograms for Figure 5 in the main manuscript

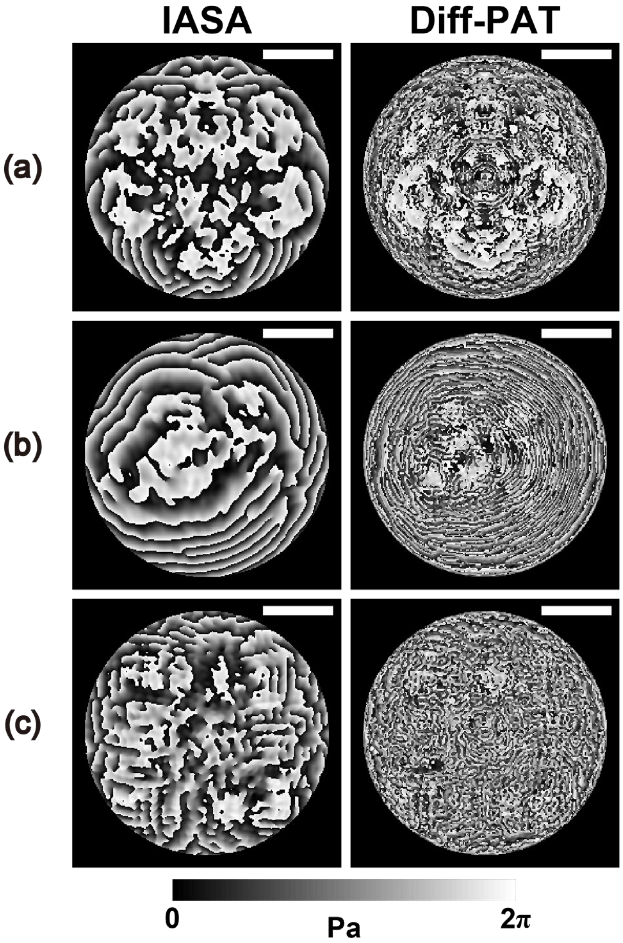

Supplementary Figure 4: Corresponding acoustic hologram for IASA and Diff-PAT in Fig. 5 in the main manuscript. The scale bar shows 10 mm. Image created using MATLAB R2020a Update 4 (https://www.mathworks.com/downloads/) and Adobe Illustrator 24.2.1 (https://www.adobe.com/products/illustrator.html).

Supplementary Figure 4 shows the acoustic holograms attached to the transducer side. These acoustic holograms propagate, and project image as shown in Figure 5 in the main manuscript.

6. Evaluating the Manufacturing Tolerance of Acoustic Hologram

By inspecting acoustic holograms generated by Diff-PAT in Supplementary Figure 4, we observe more high frequency components than acoustic hologram by IASA. These high frequency components need to translate well into the manufactured phase plates, and the effect of manufacturing tolerance to the acoustic hologram was determined. Manufacturing tolerance was assumed to be normally distributed with mean

($\mu = 1$), and the standard deviation (σ) was varied between 0 to 0.3. The results are as shown in Supplementary Figure 5, and PSNR drops by 3 dB when the $\sigma \approx 20\%$. If this manufacturing tolerance were to be issue, low-pass filter or weighted regularization value such as sum of spatial gradient can be added to reduce the high frequency components.

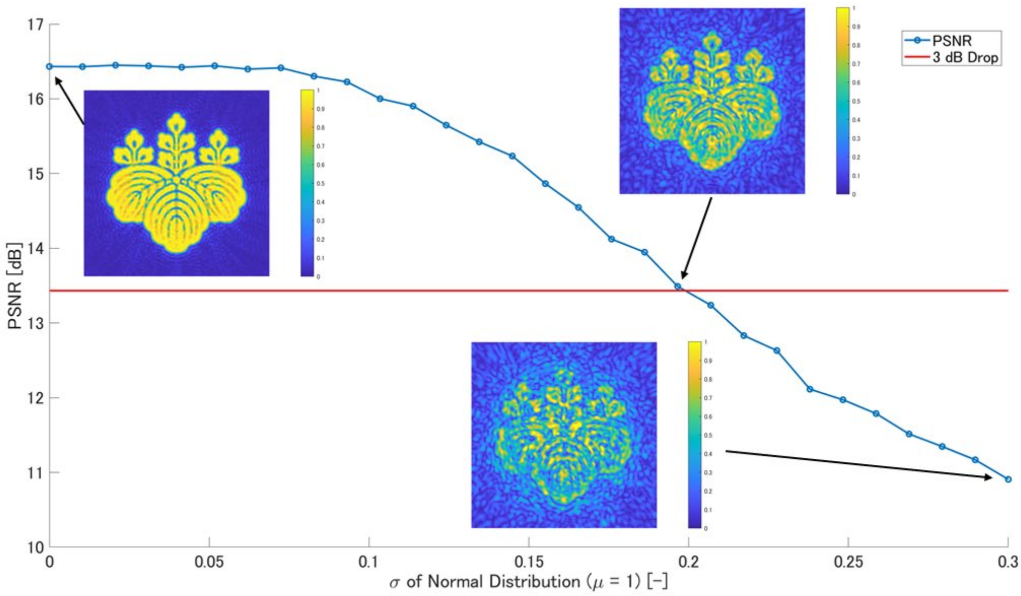

Supplementary Figure 5: Evaluating the tolerance of acoustic hologram to manufacturing accuracy. Image created using MATLAB R2020b Update 2 (https://www.mathworks.com/downloads/) and Microsoft Office PowerPoint Office 365 (https://www.microsoft.com/en-ww/microsoft-365/powerpoint).

7. Diffraction of Acoustic Waves in Phase Plates

As shown in the Supplementary Figure 6, the Diff-PAT achieves high accuracy within the region of interest by diffracting the acoustic waves outside the field of view in loss function.

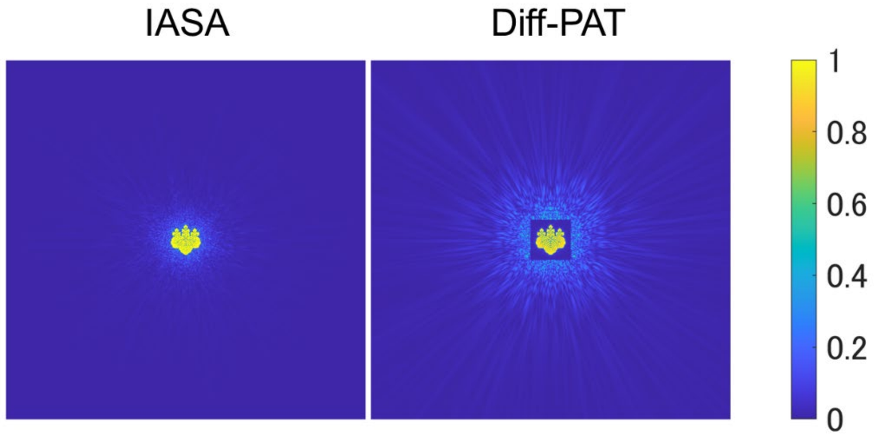

Supplementary Figure 6: Diffraction of acoustic waves outside the region of interest in Diff-PAT. Image created using MATLAB R2020a Update 3 (https://www.mathworks.com/downloads/) and Microsoft Office PowerPoint Office 365 (https://www.microsoft.com/en-ww/microsoft-365/powerpoint).
